# Evolution and functional analysis of the *GRAS* family genes in six Rosaceae species

**DOI:** 10.1186/s12870-022-03925-x

**Published:** 2022-12-06

**Authors:** Yibo Bai, Hui Liu, Kaikai Zhu, Zong-Ming Cheng

**Affiliations:** 1 College of Horticulture, Nanjing Agricultural University, Nanjing, 210095 Jiangsu China; 2 Co-Innovation Center for Sustainable Forestry in Southern China, Nanjing Forestry University, Nanjing, 210037 Jiangsu China

**Keywords:** *GRAS* genes, Rosaceae species, Duplications, Abiotic stress, Woodland strawberry

## Abstract

**Background:**

*GRAS* genes formed one of the important transcription factor gene families in plants, had been identified in several plant species. The family genes were involved in plant growth, development, and stress resistance. However, the comparative analysis of *GRAS* genes in Rosaceae species was insufficient.

**Results:**

In this study, a total of 333 *GRAS* genes were identified in six Rosaceae species, including 51 in strawberry (*Fragaria vesca*), 78 in apple (*Malus domestica*), 41 in black raspberry (*Rubus occidentalis*), 59 in European pear (*Pyrus communis*), 56 in Chinese rose (*Rosa chinensis*), and 48 in peach (*Prunus persica*). Motif analysis showed the VHIID domain, SAW motif, LR I region, and PFYRE motif were considerably conserved in the six Rosaceae species. All *GRAS* genes were divided into 10 subgroups according to phylogenetic analysis. A total of 15 species-specific duplicated clades and 3 lineage-specific duplicated clades were identified in six Rosaceae species. Chromosomal localization presented the uneven distribution of *GRAS* genes in six Rosaceae species. Duplication events contributed to the expression of the *GRAS* genes, and *Ka/Ks* analysis suggested the purification selection as a major force during the evolution process in six Rosaceae species. *Cis*-acting elements and GO analysis revealed that most of the *GRAS* genes were associated with various environmental stress in six Rosaceae species. Coexpression network analysis showed the mutual regulatory relationship between *GRAS* and *bZIP* genes, suggesting the ability of the *GRAS* gene to regulate abiotic stress in woodland strawberry. The expression pattern elucidated the transcriptional levels of *FvGRAS* genes in various tissues and the drought and salt stress in woodland strawberry, which were verified by RT-qPCR analysis.

**Conclusions:**

The evolution and functional analysis of *GRAS* genes provided insights into the further understanding of *GRAS* genes on the abiotic stress of Rosaceae species.

**Supplementary Information:**

The online version contains supplementary material available at 10.1186/s12870-022-03925-x.

## Background

Transcription factors (TFs) as regulatory proteins, act with promoter *cis*-acting elements or interact with functional regions of other transcription factors to regulate gene transcription and expression [1]. The main TF family in the plant was first identified in *Arabidopsis* [2]. In the following years, transcription factors in other plants were identified gradually with the development of the genome era [3]. Several studies had shown that transcription factors play an important role in various regulation processes of plants [4, 5].

As a transcription factor, GRAS genes participated in plant growth and development [6, 7], stress resistance [8, 9], and other life activities [10, 11]. *GRAS* originated from the three earliest members which were functionally researched, namely, gibberellic acid insensitive (GAI), scarecrow (SCR), and repressor of ga1-3 (RGA) [12–14]. In general, GRAS proteins consist of a variable N-terminal domain and a relatively conserved C-terminal domain. The C-terminal contains five extremely conserved fragments: LRI (Leucine-rich regions I), VHIID, LRII (Leucine-rich regions II), PFYRE, and SAW [15–17]. The VHIID region between LRI and LRII was a conserved domain, which was present in almost all GRAS proteins [18]. Related studies had shown that the LRI-VHIID-LRII pattern may be involved in the binding of proteins to nucleic acids or the binding of other proteins [19, 20]. In addition, the motifs of PFYRE and SAW were identified as related to GRAS protein structural integrity [21].

At present, the function of *GRAS* genes had been identified in several plant species. The number of 32 *GRAS* were found in *Arabidopsis* [22], 57 in rice [23], 54 in tomato [24], 48 in Chinese cabbage [25], 48 in physic nut [26], 62 in barely [27], and 117 in soybean [28]. Further phylogenetic tree analyses of multi-species divided GRAS proteins into 10 subfamilies, namely, DELLA, AtLAS, AtSHR, AtPAT1, AtSCL3, SCL4/7, HAM, LISCL, AtSCR, and DLT [29]. These *GRAS* genes from diverse subfamilies had been proven to be involved in various physiological processes in plants. For instance, *OsMOC1*, *SlyLs*, and *AtLAS* were reported to be mainly related to the axillary formation [30–32]. Two *Arabidopsis* GRAS proteins, *AtSCR* and *AtSHR*, positively regulated the formation of root and shoot radial patterns [33, 34]. *AtSCL21* and *AtPAT1* were mainly involved in the signal transduction of etiolated to photomorphogenic in *Arabidopsis* [35, 36]. Furthermore, several previous studies had shown that *GRAS* genes participated in plant abiotic stress processes by interacting with *bZIP* genes [37]. DELLA proteins had been proven to be involved in ABA signaling and enhanced plant tolerance to drought by interacting with the abscisic acid-responsive transcription factor ABF2 [38]. *AtSCL14* was shown to interact with the *bZIP* family gene *TGA* to activate the broad-spectrum detoxification network [39].

Rosaceae, one of the most economically valuable families, was classified into four subfamilies: Spiraeoideae, Maloideae, Rosoideae, and Amygdaloideae [40]. Rosaceae plants include economical ornamental flowers, numerous fruit species, herbs, nuts, and woody plants [41]. Although the *GRAS* family has been studied in fruit species, such as apple [42] and strawberry [43], the comparison between *GRAS* family species in Rosaceae had not been reported. In this study, we selected the representative plants from the four subfamilies of Rosaceae, including woodland strawberry, black raspberry, Chinese rosa, European pear, peach, and apple. The members of the *GRAS* family were identified in six Rosaceae species, comprehensive phylogenetic analysis, motif analysis, gene duplication, *Ka/Ks* analysis, and *cis*-acting element analysis were executed. Combining transcriptome analysis and RT-qPCR analysis, we determined the responses of *GRAS* family members to drought and salt stress in woodland strawberry. This study may serve as the basis for revealing the evolutionary relationship of the *GRAS* family in plant growth and development.

## Materials and methods

### Identification and classification of the *GRAS* family

To identify the *GRAS* genes of six Rosaceae species, all protein-coding sequences of *Malus domestica* (v1.0.a1), *Rubus occidentalis* (v3.0), *Pyrus communis* (v2.0), *Fragaria vesca* (v1.1.a2), *Prunus persica* (v2.0.a1), and *Rosa chinensis* (v1.0) were downloaded from the Genome Database for Rosaceae (GDR) site (https://www.rosaceae.org/). The hidden Markov models (HMMs) of the *GRAS* (PF03514) were downloaded from Pfam 35.0 (http://pfam.xfam.org/), and used to investigate putative *GRASs* with an e-value cutoff < 1.0 using HMMER v3.1 [44]. To validate the accuracy of the possible *GRASs*, all candidate *GRAS* genes were examined and analyzed by Pfam v35.0 and SMART v9.0 (http://smart.embl-heidelberg.de/) [45].

Multiple amino acid alignments of GRAS proteins were aligned and the phylogenetic tree was constructed with the maximum likelihood method and 1000 bootstrap replicates using IQ-TREE v2.1.3 in six Rosaceae species [46]. The Evolview tool was used to improve graphical presentations of the trees [47]. In the clade with bootstrap values larger than 50, the lineage-specific duplications were defined as the *GRAS* genes appeared in two or more Rosaceae species, and the species-specific duplications occurred in one Rosaceae species [48]. The protein sequences of the *GRAS* gene in six Rosaceae species and *Arabidopsis* were aligned by the MUSCLE program in MEGA X and the maximum-likelihood tree was built using IQ-TREE v2.1.3.

### Conserved motif analysis of the *GRAS* genes

The putative motifs in six Rosaceae species *GRAS* family were predicted by the MEME website (https://meme-suite.org/meme/). The parameter was set according to described previously [49].

### Chromosomal location and gene duplication

The chromosomal locations and lengths of the *GRASs* were obtained from the genome database. Then, the *GRAS* genes were represented graphically with MapChart. All *GRAS* protein sequences were searched against themselves by NCBI-BLAST 2.13.0 + [50], and duplication events were investigated using DupGen_finder [51].

### Calculation of *Ks*, *Kax/Ks* values

To calculate the selection pressure of *GRAS* genes in six Rosaceae species, the *Ks* (synonymous substitutions) and *Ka/Ks* (nonsynonymous to synonymous substitution) ratios of gene pairs were calculated using MEGA X software and Perl script. *Ka/Ks* > 1 indicated positive selection, *Ka/Ks* = 1 suggested neutral selection, and *Ka/Ks* < 1 implied purification selection [52].

### *Cis*-acting elements in the *GRAS* promoter regions

The 1500 bp upstream sequences from the promoters of *GRAS* genes in six Rosaceae species were extracted from the assembly file using Perl script. The *cis*-acting elements in the putative promoter regions were analyzed by PlantCARE (http://bioinformatics.psb.ugent.be/webtools/plantcare/html/) [53].

### GO functional analysis of *GRAS* genes

To report the predicted functions of GRAS proteins, the GO terms for *GRASs* in six Rosaceae species were collected from the Gene Ontology Consortium (http://geneontology.org) [54].

### Transcriptome analyses of woodland strawberry *GRAS* genes

To identify tissue-specific expression patterns of *GRAS* genes in woodland strawberry, RNA-Seq data were collected from Strawberry Genome Resources (http://bioinformatics.towson.edu/strawberry) [55]. The datasets of PRJNA733854 and PRJNA472896, which involved strawberry gene expression upon drought and salt stress, were downloaded from the NCBI website (https://www.ncbi.nlm.nih.gov) [56, 57]. The FPKM (fragments per kilobase million) values were calculated by R v4.0.2. The ‘scale’ parameter was used to normalize transcriptome data. The expression levels of *GRAS* genes were visualized using the ComplexHeatmap package (v2.4.3) in the R (v4.0.2).

### STEM analysis of *GRAS* genes in woodland strawberry

The DEGs were classified into different clusters according to time sequence profile analysis by Short Times-series Expression Miner (STEM) software [58].

### Co-expression network of *GRAS* with *bZIP* genes in woodland strawberry

The FPKM values of *GRAS* and *bZIP* genes were extracted from the drought and salt stress transcriptomes. The Pearson correlation coefficients (PCCs) between *GRAS* and *bZIP* genes were calculated and constructed in a co-expression network using Cytoscape_v3.7.2 (PCCs ≥ 0.6, *P* < 0.05).

### Plant material stress treatment, RNA extraction and RT-qPCR analysis

Woodland strawberry plants (*Fragaria vesca*, Hawaii4) were grown in greenhouse at Nanjing Agricultural University (Jiangsu, China), and maintained at room temperature under a photoperiod of 16/8 h. Two-month-old plants were used for drought and salt treatment. Tender (the stage before fully expanded mature leaves) and old (the stage of fully expanded mature leaves) leaf samples were collected at 0, 3, 5, and 7 days after water was withheld. In addition, strawberry leaves were used as salt stress treatment and samples were collected at 0, 3, 5, and 7 days after treatment.

Total RNA was extracted using the Plant Total RNA Isolation Kit Plus (Foregene, Chengdu, China). First strand cDNA was synthesized using PrimerScript™ RT reagent Kit with gDNA Eraser (TaKaRa, Dalian, China). RT-qPCR analysis was performed by Light Cycler 480 II (Roche, Switzerland) with TB Green® Premix Ex Taq™ (TaKaRa, Dalian, China). 18sRNA was used as an internal reference gene to normalize the expression of *GRAS* genes using the 2^−△△Ct^ method [59]. Each set of RT-qPCR data was calculated with three replicates. The primer sequences used for RT-qPCR were listed in Table S5.

## Results

### Identification and characterization of the *GRAS* family in six Rosaceae species

After excluding the redundant sequences, a total of 333 *GRAS* genes were identified. For the six species, strawberry, apple, black raspberry, pear, Chinese rosa and peach, 51, 78, 41, 59, 56 and 48 *GRAS* genes were detected, respectively (Table 1). The apple (78) had the maximum number and highest proportion of *GRAS* genes compared with the other five species. The number of *GRAS* genes in the other five species was similar.

**Table 1:**
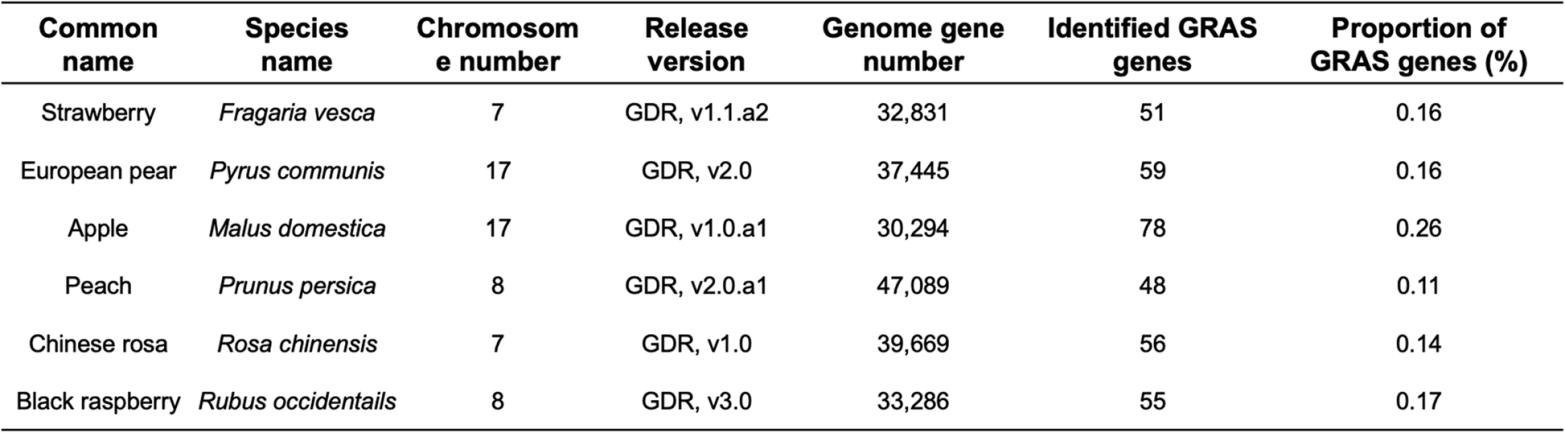
Number of *GRAS* genes in six Rosaceae species

### Phylogenetic analysis of GRAS protein

**Fig. 1:**
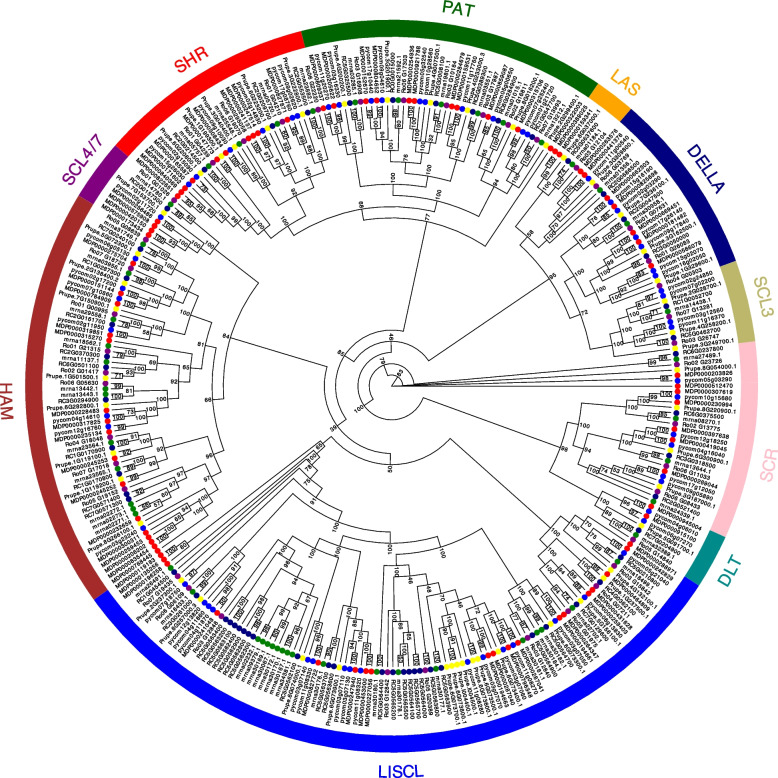
Phylogenetic tree of GRAS protein from Chinese rosa, European pear, peach, apple, black raspberry, and strawberry. The 333 *GRASs* can be divided into ten groups (PAT1, HAM, SCR, DLT, LAS, SCL3, SHR, SCL4/7, LISCL, and DELLA)

To analyze the phylogenetic relationships of GRAS proteins in six species. The GRAS sequences were used to construct a maximum likelihood tree by IQ-TREE. *Arabidopsis* was used as an outgroup, and the *GRAS* genes of six Rosaceae species were divided into ten subfamilies, including PAT1, SHR, SCL, HAM, DELLA, SCL3, LAS, SCL4/7, DLT, SCR (Fig. 1 and Fig. S2). In addition, the clades were shaped by bootstrap values larger than 50 in six Rosaceae species. The 301 *GRAS* genes (appearing in one Rosaceae species) were identified as 15 species-specific duplicated clades, and the 3 lineage-specific duplicated clades, including 12 *GRAS* genes appeared in two or more Rosaceae species (Fig. S1).

### Conserved motif analysis of GRAS protein

**Fig. 2:**
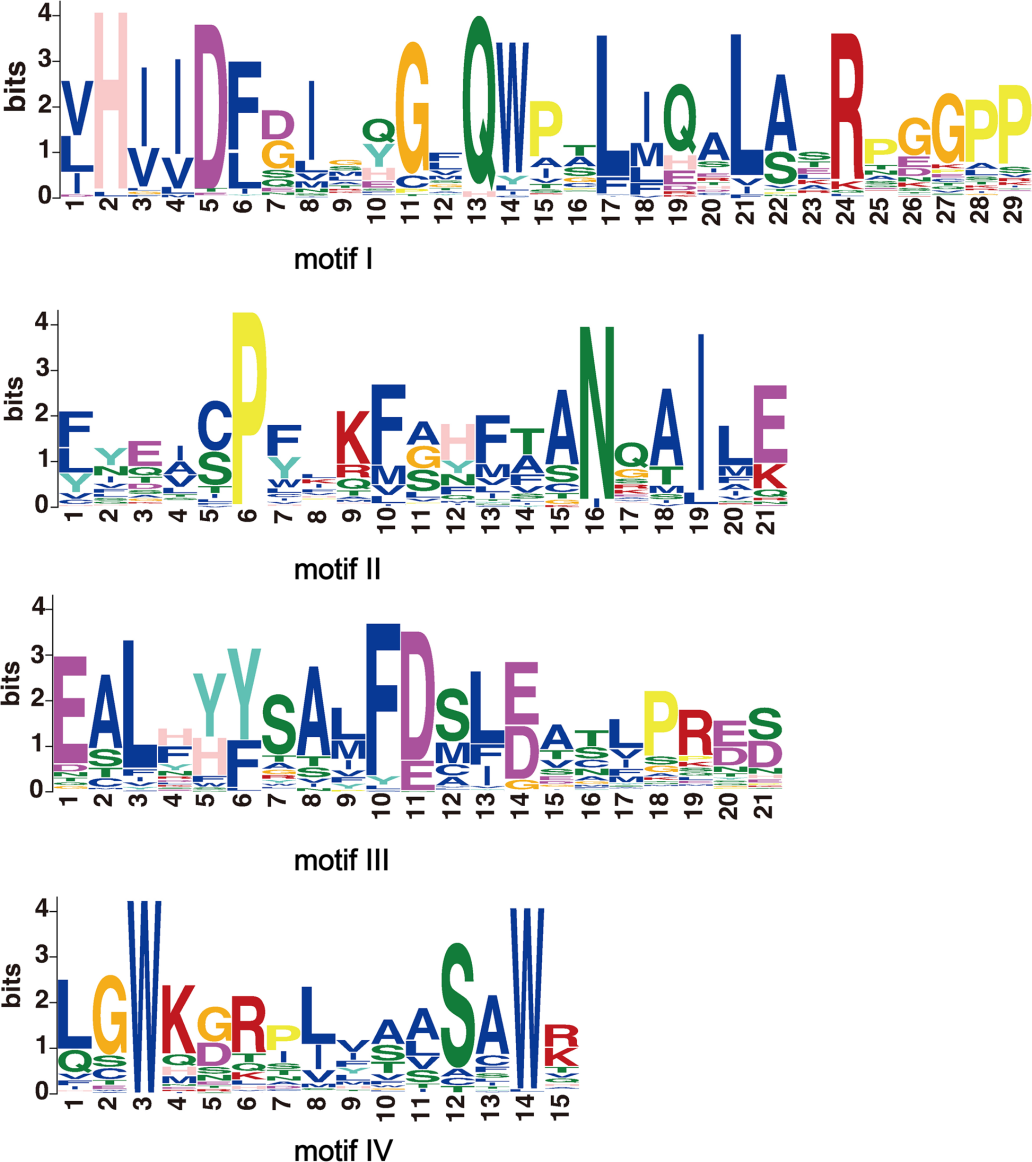
Analysis of four conserved motifs of GRAS proteins in six Rosaceae species: *Rubus occidentails*, *Rosa chinensis, Prunus persica, Malus domestica, Pyrus communis,* and *Fragaria vesca*

The unique motif sequences provided insight into protein function, in order to identify motif constructions of the GRAS proteins, we submitted 333 *GRAS* predicted amino acid sequences to the MEME website. A total of 20 motifs were used as the number of queries for the MEME program. Figure 2 showed the four conserved sites in six Rosaceae species, including the VHIID domain, SAW motif, LRI region, and PFYRE motif. This result suggested the highly conserved *GRAS* gene family.

### Chromosomal distribution and duplication events of the *GRAS* genes

The *GRAS* genes were distributed unevenly across the chromosomes in six Rosaceae species (Figs. S3, S4, S5, S6, S7 and S8). There was even no *GRAS* gene distribution on the chromosomes of some species, for instance, no *GRAS* gene was found on chromosome 16 in apple (Fig. S4), chromosomes 8 and 14 in pear (Fig. S6), and chromosome 4 in woodland strawberry (Fig. S3). In addition, chromosome 5 had the largest number (26) and highest proportion (47%) of the *GRAS* gene detected in Rosa (Fig. S7).

Gene duplication, as one of the main ways of multigene family expansion, was ubiquitous in plant species [60]. In this study, we identified the number of tandem (TD), whole-genome (WGD), proximal (PD), transposed (TRD) and dispersed (DSD) duplication genes in the *GRAS* gene family of six Rosaceae species (Additional file 11: Table S3). WGD and TRD events were found in all six Rosaceae species, and 72.88% (30/59) of *GRAS* genes were identified in pear higher than others in WGD events. TD events occurred in apple, pear, peach, rosa, and strawberry. The largest proportion was 19.23% (15/78) in apple compared to the other five Rosaceae species. The 198 DSD events were identified with 130 *GRAS* genes in apple, peach, rosa, black raspberry, and strawberry. PD events only appeared in rosa, apple, and strawberry.

### *GRAS* gene evolutionary in six Rosaceae species

**Fig. 3:**
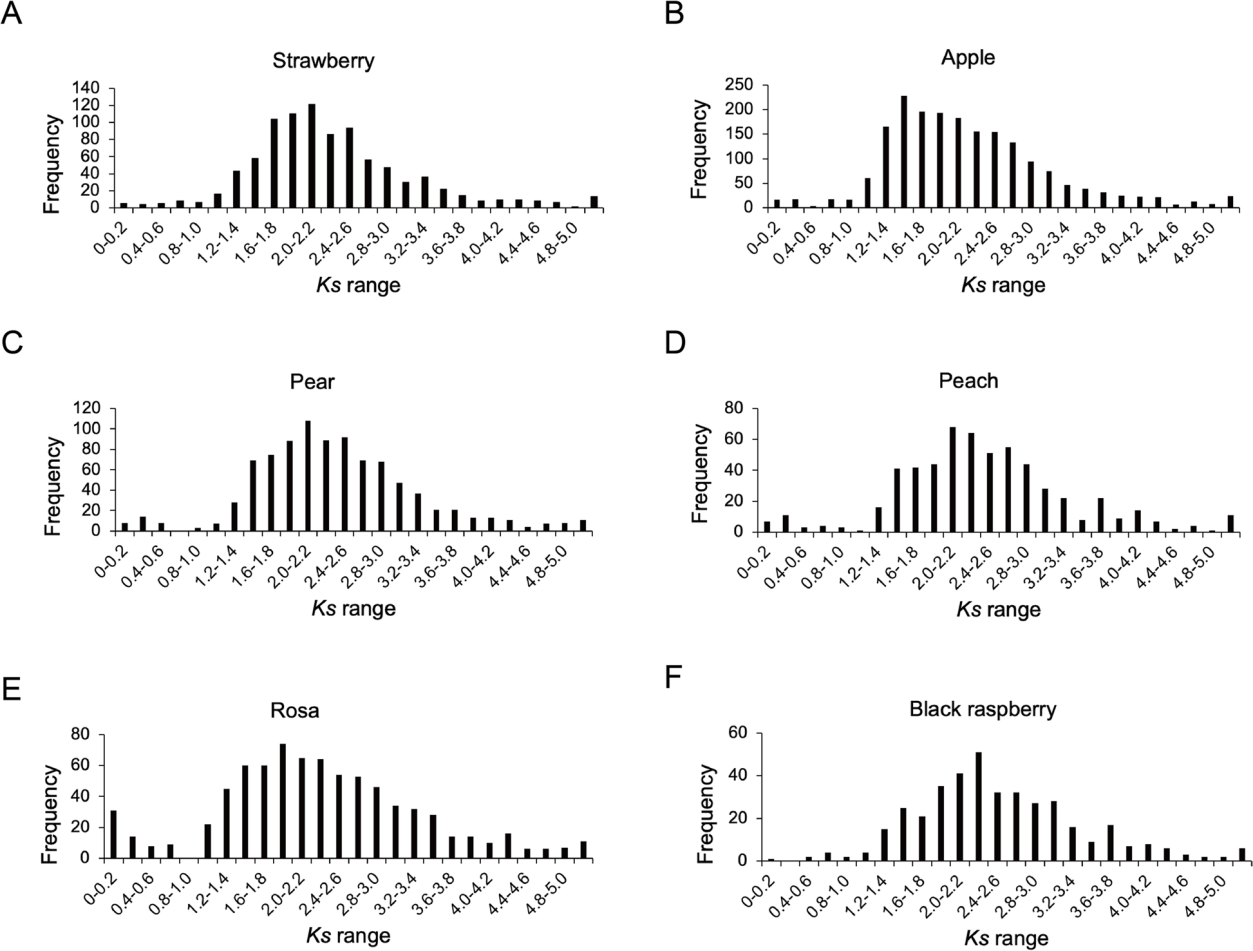
The *Ks* values of *GRAS* genes in six Rosaceae species

The synonymous (*Ks*) represented the amino acid sequence remaining unchanged after the mutation [61], and the values of *Ks* in each gene pair were calculated among six Rosaceae species using Perl script. The value of *Ks* was mainly distributed at 1.2 to 3.8 in apple, strawberry, pear, black raspberry, and peach (Fig. 3). Compared with the other five species, the main distribution of *Ks* values in rose was 1.0 to 4.4 (Fig. 3E). In apple, the Ks value peaked in the 1.4–1.6 region. The other three Rosaceae species, woodland strawberry, pear, peach with the peak of *Ks* values between 2.0–2.2 and showed a similar pattern (Fig. 3A, C and D). These results suggested the relatively recent duplication of *GRAS* genes in apple.

**Fig. 4:**
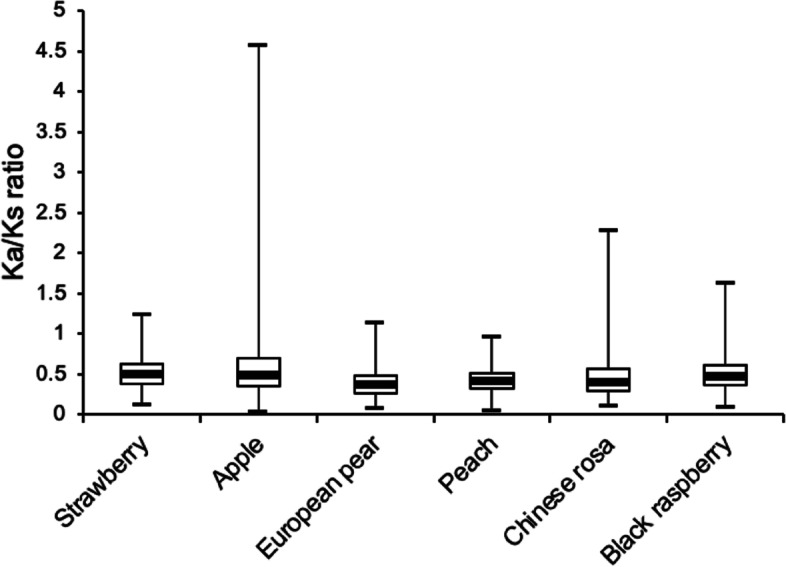
The *Ka*/*Ks* values of *GRAS* genes in six Rosaceae species

The nonsynonymous substitutions (*Ka*) and synonymous (*Ks*) ratio of two protein-coding genes were determined whether the selective pressure acted on the gene [62]. In this study, the *Ka/Ks* value of 97.7% (5422/5547) of gene pairs was less than 1, indicating that the gene pairs were under purifying selection. The 2.1% (117/5547) of gene pairs had a ratio greater than 1, which implied a positive selection of the gene pairs (Fig. 4 and Table S4).

### Functional prediction of *GRAS* genes in six species

**Fig. 5:**
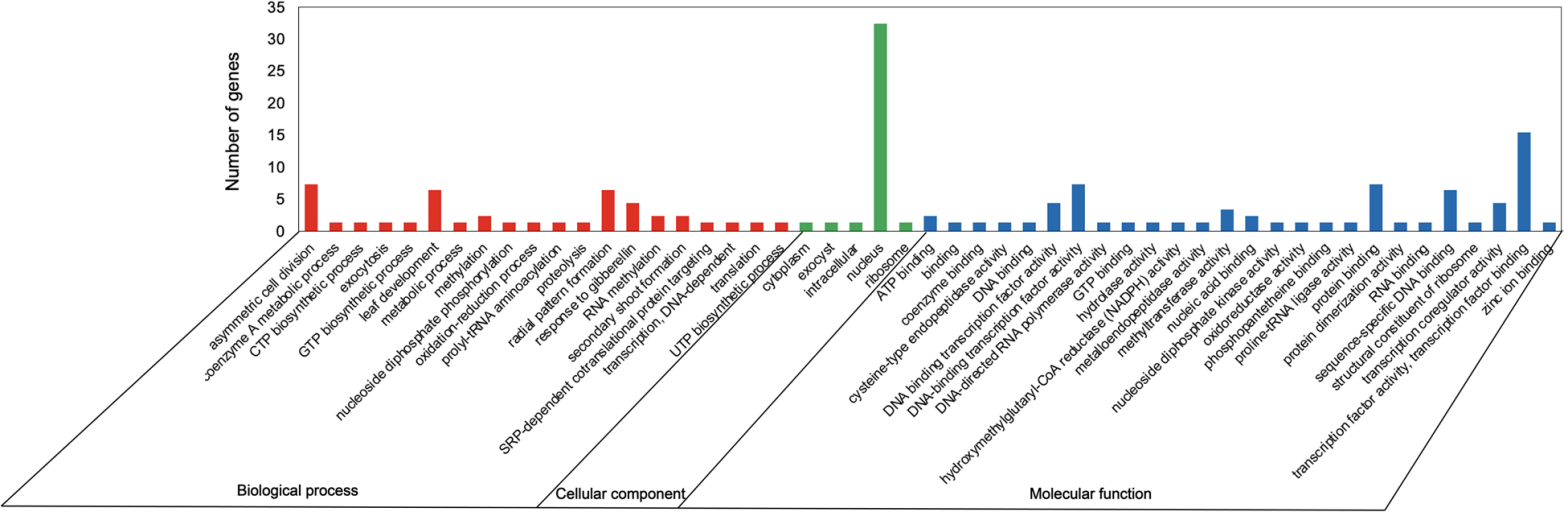
Gene ontology (GO) analyses of *GRAS* genes in six Rosaceae species

GO annotations of 333 *GRAS* genes were assigned and classified into three categories: molecular function, biological process, and cellular component (Fig. 5). The results of the biological process showed that the terms asymmetric cell division (GO:0008356), leaf development (GO:0048366), radial pattern formation (GO:0009956), and response to gibberellin (GO:0009739) were enriched. The top GO term in the cellular component was nucleus (GO:0005634). According to the molecular function results, most *GRAS* genes were associated with transcription factor activity (GO:0003700 and GO:0000989). These results suggested that *GRAS* genes were mainly expressed in the nucleus as transcription factors and functioned to regulate plant development and respond to hormone-related stress. In addition, 5609 *cis*-acting elements for all the *GRAS* gene promoter regions were predicted in six Rosaceae species (Fig. S9). These elements were divided into light responsive, hormone response, metabolism regulation, defense and stress response according to the functions. Elements related to stress occupied a high proportion, including the elements involved in the low-temperature response, drought, defense, and stress response. Many hormone-related responses were also reflected in *cis*-acting elements, such as auxin, salicylic acid, jasmonic acid, abscisic acid, and gibberellin responses.

### Expression patterns of woodland strawberry *GRAS* genes

**Fig. 6:**
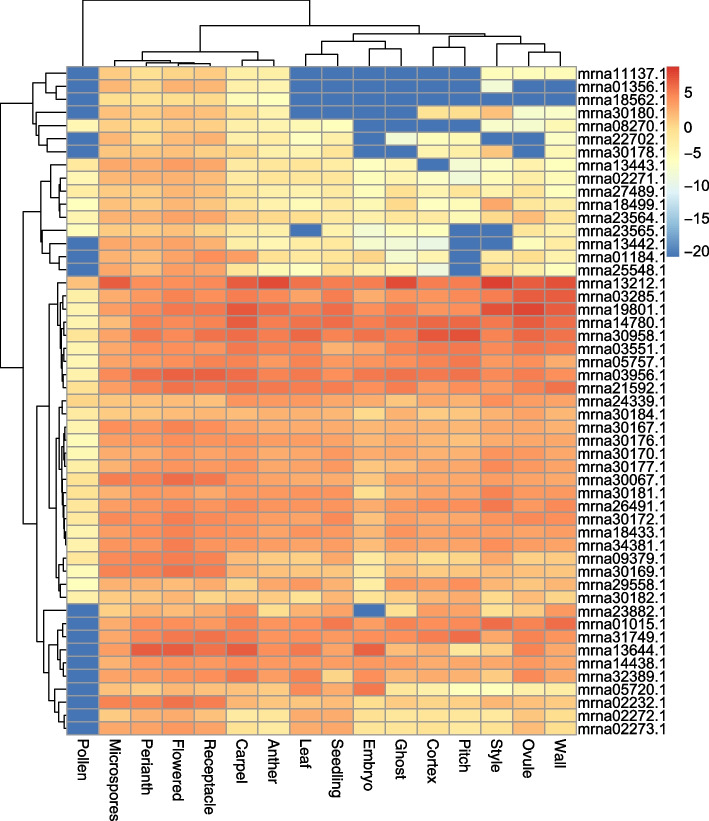
Heatmap shown the expression of *GRAS* genes in 42 tissues and developmental stages of woodland strawberry

We collected the expression patterns of the strawberry *GRAS* gene family in different tissues, including pollen, microspores, perianth, flowered, receptacle, carpel, anther, leaf, seeding, embryo, ghost, cortex, pitch, style, ovule, and wall. The heatmap showed that at least two-thirds of the genes were highly expressed in almost all tissues (Fig. 6). Among them, the *GRAS* genes in woodland strawberry were expressed at a low level in pollen. Additionally, the *GRAS* genes of strawberry had similar expression patterns in the carpel, anther, leaf, seedling, embryo, ghost, cortex, pitch, style, ovule, and wall. These results implied that *GRAS* genes have participated in the growth and development of woodland strawberry.

### Transcriptome and RT-qPCR expression analysis of *FvGRASs* under abiotic stress

**Fig. 7:**
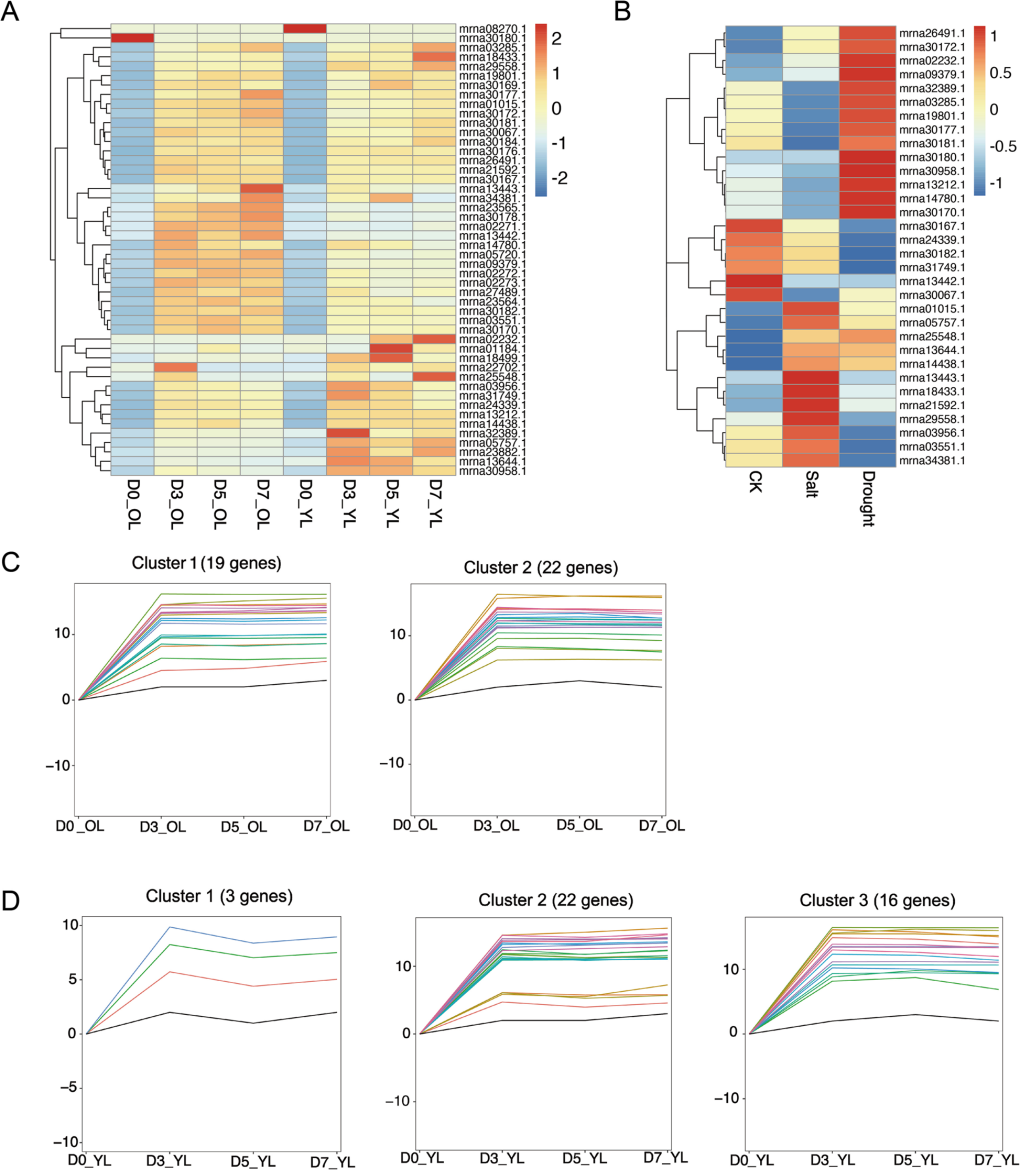
Heatmap shown the expression of *GRAS* genes under drought (**A**) and salt stress (**B**) in woodland strawberry. **C** Temporal changes of *GRAS* genes under drought stress in woodland strawberry old leaves. **D** Temporal changes of *GRAS* genes under drought stress in woodland strawberry young leaves

To verify the role of *FvGRAS* under drought stress, we analyzed the expression pattern of woodland strawberry under drought treatment. After drought treatment, the old leaves and young leaves were clustered at 0d, 3d, 5d, and 7d (Fig. 7A). Heatmap showed that at least half of the *GRAS* genes were significantly increased in expression upon drought stress. The *GRAS* genes were divided by STEM analysis, in which two and three profiles had *P* < 0.01 in old (Fig. 7C) and young leaves (Fig. 7D), respectively. The expression levels of *FvGRASs* were gradually increasing under drought stress. In strawberry fruit, we found that at least one-third of the *GRASs* responds to drought and salt stress. Interestingly, the genes differed between salt and drought stress (Fig. 7B), such as *mrna30170.1* was highly expressed in drought stress but lower upon salt stress, which indicated that the *GRAS* family was related to the resistance of strawberry to drought stress.

**Fig. 8:**
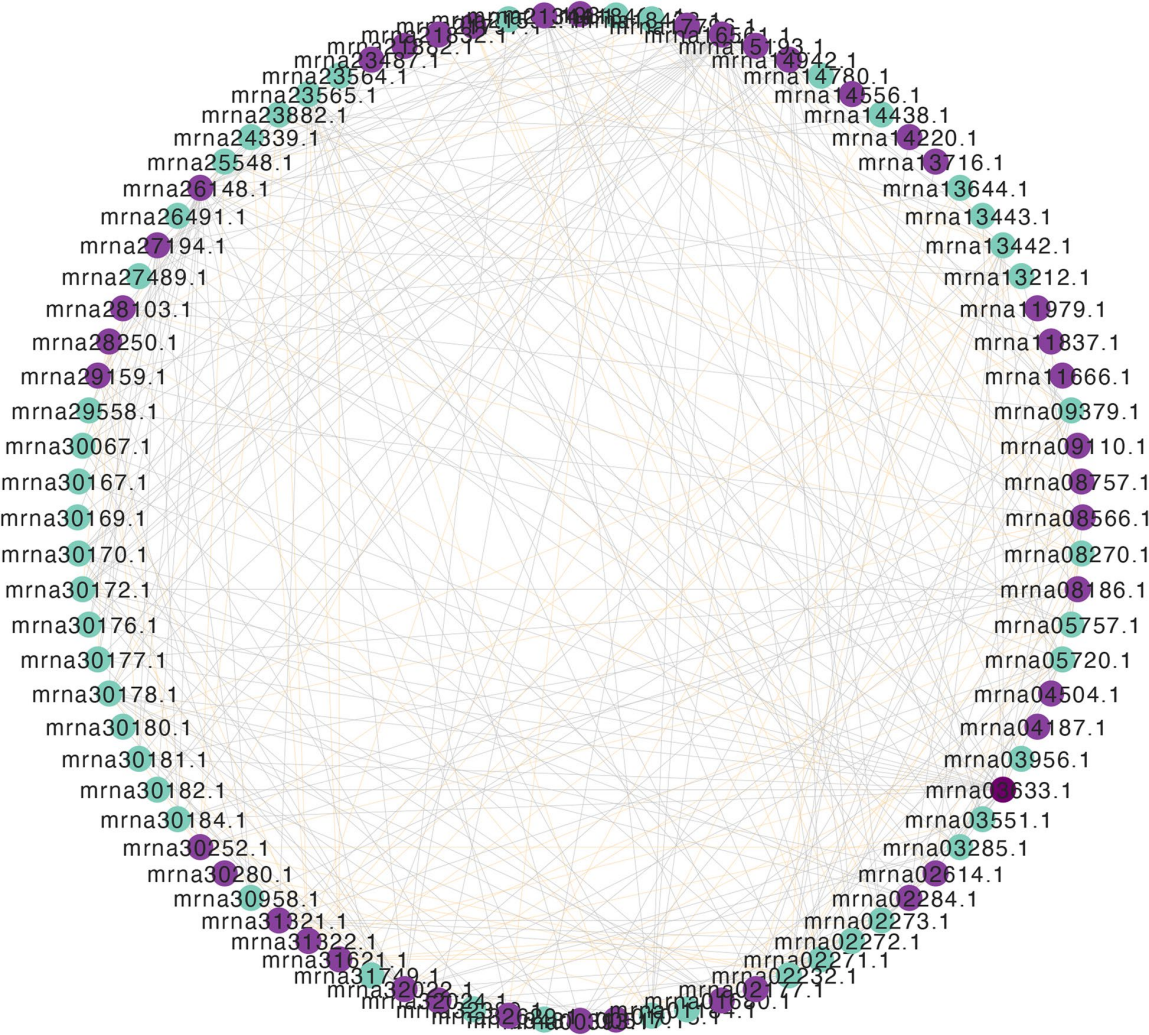
The correlation analysis between *GRAS* and *bZIP* genes in response to salt and drought treatments. Different edge line colors represent either positive (orange) or negative (grey) correlations

The values of PCC were calculated to predict the interaction of *GRAS-bZIP* in woodland strawberry. 91 *GRAS-bZIP* gene pairs were significantly positively correlated with expression under drought and salt stress (Fig. 8). 255 *GRAS-bZIP* gene pairs showed negative correlations. These significantly correlated gene pairs may be involved in the activities of response to abiotic stress in strawberry.

**Fig. 9:**
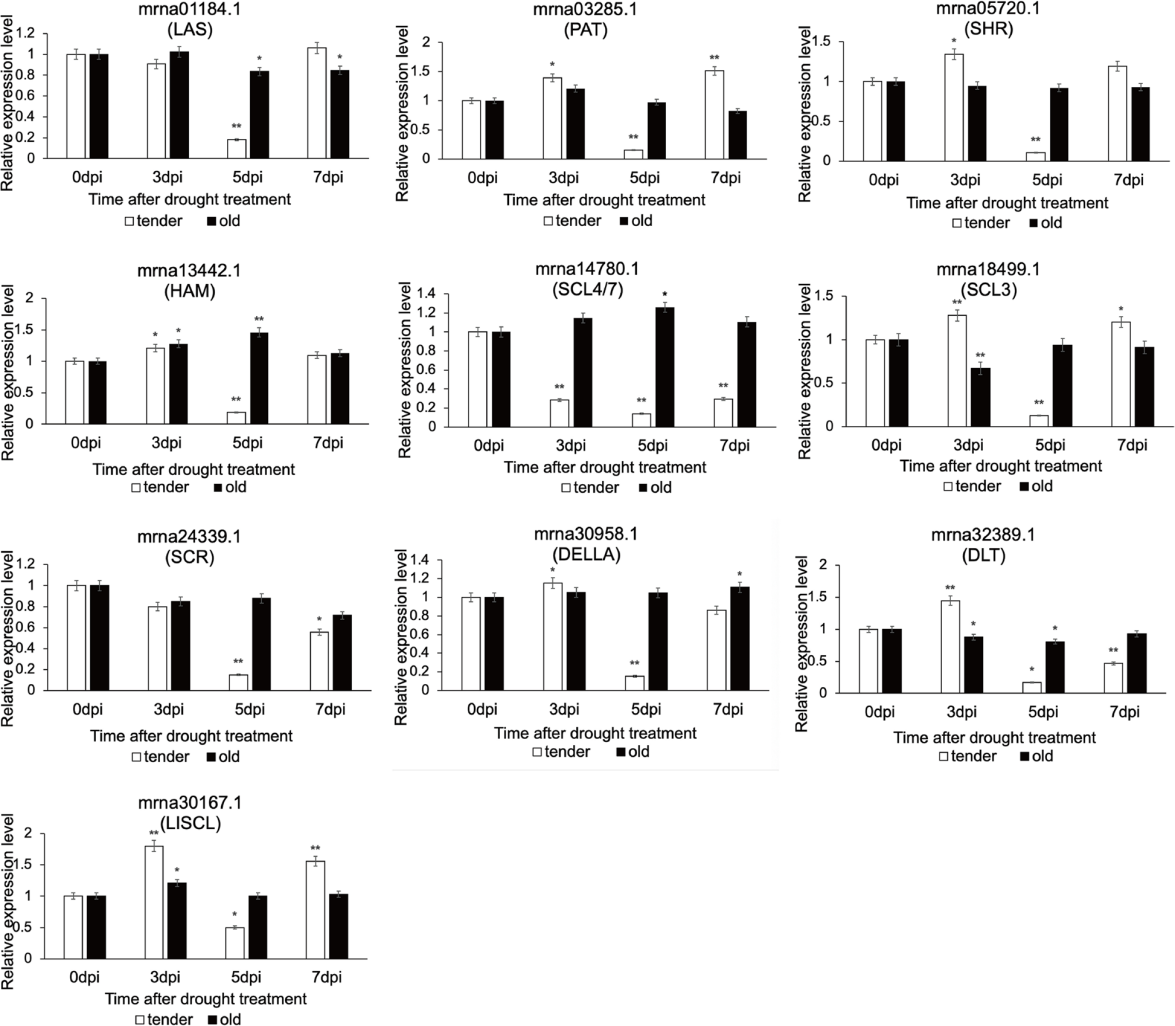
Relative expression levels of *GRAS* genes in woodland strawberry under drought stress

Subsequently, according to the classification of different subfamilies, we used RT-qPCR to detect the expression levels of genes in subfamilies (Fig. 9). In the tender leaf tissues under drought stress, the gene expression levels of almost all subfamilies showed a decline and then ascending trend. On the fifth day after treatment, the expression of *FvGRAS* genes was the lowest in young leaves. Interestingly, the expression levels of *mrna14780.1* (SCL4/7 subfamily) and *mrna24339.1* (SCR subfamily) were low in tender leaves at different treatment stages. We speculated that these subfamilies may not be involved in the response to drought stress in the young strawberry leaves. In addition, in the old leaves, with the extension of the treatment time, the gene expression trend was slower compared to the tender leaves. For instance, the relative expression levels of LAS (*mrna01184.1*), PAT (*mrna03285.1*), SHR (*mrna05720.1*), SCL3 (*mrna18499.1*), and LISCL (*mrna30167.1*) subfamily genes were lower than tender leaves at 7d in old leaves. These results suggested that *FvGRAS* families respond to drought stress and that the responses in diverse areas seemed to be different.

**Fig. 10:**
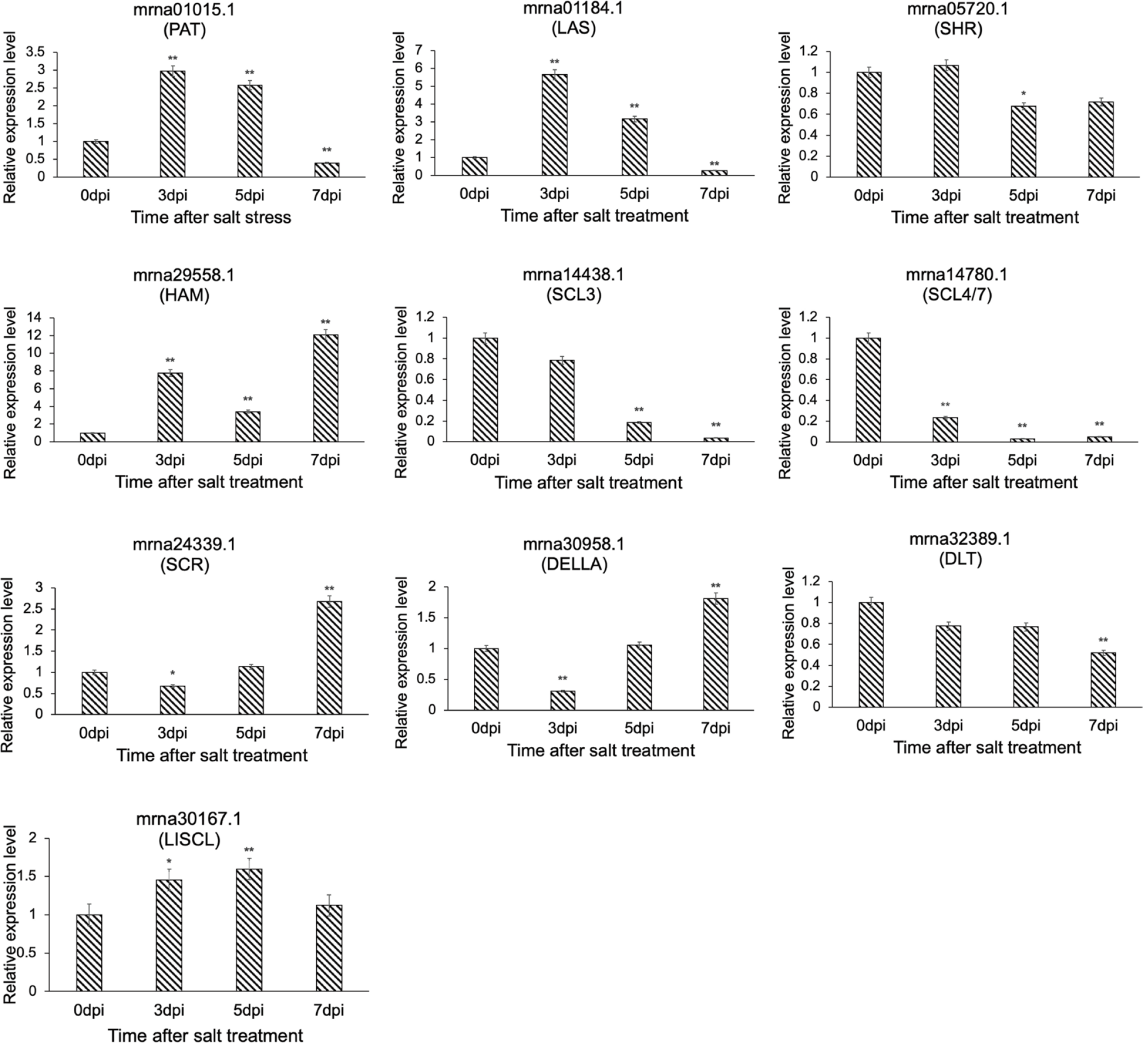
Relative expression levels of *GRAS* genes in woodland strawberry under salt stress

To further verify the role of the *FvGRAS* gene family in other abiotic stresses, we detected the relative expression level of the *FvGRAS* gene after salt treatment (Fig. 10). These results implied that the expression levels of the *FvGRAS* genes under salt stress were obviously higher than drought stress. The expression of PAT (*mrna01015.1*), LAS (*mrna01184.1*), HAM (*mrna29558.1*), and SCR (*mrna24339.1*) subfamily genes was more obvious. Interestingly, we observed that genes of different subfamilies were highly expressed at different treatment periods, for instance, *mrna01015.1* (PAT) and *mrna01184.1* (LAS) were mainly highly expressed on days 3 and 5 after treatment, while *mrna29558.1* (HAM), *mrna24339.1* (SCR), and *mrna30958.1* (DELLA) were mainly highly expressed on day 7 after treatment. In addition, SCR (*mrna24339.1*) and DELLA (*mrna30958.1*) subfamily genes increased with the treatment time, while the expression of PAT (*mrna01015.1*), LAS (*mrna01184.1*), SCL3 (*mrna14438.1*) subfamily genes decreased with the treatment time. These results indicated that *FvGRAS* genes perform unique functions and jointly regulate the resistance of plants to adversity during abiotic stress.

## Discussion

Members of the *GRAS* family played an important regulatory role in multiple biological processes [63, 64]. In rice, *OsGRAS23* was demonstrated to be involved in the drought stress response by binding to the promoters of stress-responsive genes [65]. *VaPAT1*, an inducible *GRAS* gene, has been identified to increase cold, drought, and salt tolerance in transgenic *Arabidopsis* [66]. In tomato, overexpression of *SlGRAS40* was characterized to enhance tolerance to abiotic stress [67]. In this study, 333 *GRAS* genes were characterized in six Rosaceae species. The *GRAS* genes could be divided into ten families: PAT, SHR, SCL4/7, HAM, LISCL, DLT, SCR, SCL3, DELLA, and LAS.

### Different duplication patterns drive the expansion of *GRAS* genes in six Rosaceae

Gene duplication played an important role in the formation of new functional genetic material and the creation of new species [68–70]. As a common phenomenon, there were multiple pathways for gene duplication events [71–74], and the accumulation of beneficial mutations was preserved through selective evolution [75]. As a transcription factor family, the duplication events of *GRAS* genes had been widely reported [76]. Gene duplication serves as a source of evolutionary novelty for the *GRAS* gene family in both monocotyledonous and dicotyledonous lineages [77, 78]. Studies had shown that gene duplication events facilitate the expansion of the *GRAS* gene family in rice and *Arabidopsis* [23]. In this study, five duplication events were identified in six Rosaceae species. We found a large number of tandem duplications (19.23%), PD events (100%), and DSD events (75.64%) in apple, WGD events (72.88%) in pear, and TRD events (68.29%) in black raspberry, which implied that duplication events have contributed to the expansion of the *GRAS* gene family, especially in apple.

Species-specific duplications and lineage-specific duplications measured the occurrence of duplication events in a species or species [79]. (301/333) 90.39% species-specific duplicate genes were observed, (12/333) 3.6% lineage-specific duplicate genes were observed. This result suggested that the evolution of *GRAS* genes involved the participation of lineage-specific duplications and species-specific duplications. Meanwhile, species-specific duplication promoted the expansion of the *GRAS* genes more than lineage-specific duplication in six species.

### *GRAS* genes evolved differently among the six Rosaceae species

The pairwise *Ka/Ks* ratio was an important criterion to measure the type of gene selection pressure [80]. Previous studies had demonstrated that *HrGRAS* genes involved in duplication in sea buckthorn undergo strong purifying selection pressure according to the *Ka/Ks* ratios [81]. *Ka/Ks* analysis showed that 88.2% of *GRAS* genes proceed a purifying selection in barley [27]. Similarly, in this study, as shown in Fig. 4 and Table S4, by calculating the *Ka/Ks* values of six species, we found that most of the *GRAS* genes were subjected to purification selection. The *Ka/Ks* values of all genes in peach were less than 1, indicating that all genes in peach were in a purified state, which may be beneficial to maintaining gene function and eliminating harmful mutations during evolution [82]. In addition, almost all genes in woodland strawberry and European pear had purifying selection. By further analysis of the *Ka/Ks* ratios, a higher proportion of *GRAS* genes were subject to positive selection in apple (3.86%) and black raspberry (4.29%). This result showed that apple and black raspberry had a faster evolutionary process than the other four Rosaceae species, revealing that these genes help plants resist various stresses [83].

### Different expression patterns of *FvGRASs* in response to drought and salt stress in woodland strawberry

Previous studies had shown that *GRAS* genes were involved in plant root system, axillary meristem development, shoot meristem maintenance, and gibberellin signal transduction [7, 11]. For instance, as a key factor in rice tillering, *OsMoC1* was involved in the initiation of lateral meristems and the formation and growth of tiller buds [30]. *AtSCL28* has been reported to regulate the balance of cell size and number in *Arabidopsis* [84]. The heatmap showed the expression patterns of *GRAS* genes in different tissues and developmental stages of woodland strawberry, and it was found that at least two-thirds of *GRAS* genes were highly expressed in various tissues (Fig. 5), which indicated that *GRAS* genes were involved in plant growth and development stage in woodland strawberry. However, most of the *GRAS* genes were expressed at lower levels in pollen, and we inferred that *GRAS* genes contribute less to the reproductive process in woodland strawberry.

The *GRAS* members were reported widely involved in regulating stress responses [37, 85]. The analysis of *cis*-elements in the promoter regions of *GmGRAS* genes revealed the potential regulation during saline and dehydration stresses in soybean [86]. In the present research, stress-related *cis*-elements were found in six Rosaceae species, including those for MBS, ABRE, and TC-rich repeats, indicating that *GRAS* genes may regulate drought and salt stress responses in six Rosaceae species. *GRAS* genes also participate in stress response by interacting with the *bZIP* genes in plants. In *Arabidopsis*, the GRAS regulatory protein member, SCL14 interacts with *bZIP* subfamily TGA proteins to enhance the plant defense system [39]. In this study, by analyzing previous transcriptome data, we revealed the expression patterns of *FvGRASs* under drought and salt stress conditions. The results showed that the expression levels of most *GRAS* genes were significantly changed. In addition, we calculated the correlation coefficient between *GRAS* and *bZIP* genes, and the results showed that most *GRAS* genes have significant positive or negative correlations with *bZIP* genes, indicating that *GRAS* genes may respond to abiotic stress by regulating *bZIP* genes.

To verify whether *FvGRASs* were involved in the response to abiotic stress, we designed drought and salt stress treatments. The expression levels of *GRAS* genes in tender leaves were upregulated at 3 d, and decreased at 5 d, and then were upregulated at 7 d under drought conditions. In contrast, the expression levels of *mrna24339.1* (SCR) and *mrna30958.1* (DELLA) were always up-regulated under salt stress. Interestingly, the HAM subfamily and PAT subfamily had higher expression levels under drought and salt stress that the other subgroups. These data indicated that the *FvGRAS* family was linked to the responses to drought and salt stresses in woodland strawberry, and the genes had various pathways to participate in various abiotic stresses.

## Conclusion

In this study, 51, 78, 41, 59, 56, and 48 *GRAS* genes were identified in pear, apple, black raspberry, Chinese rosa, strawberry and peach, respectively. Analysis of duplication events in six Rosaceae species revealed that the duplication modes of these six Rosaceae species were diverse, and species-specific duplications contributed more to the expansion of *GRAS* genes in the six Rosaceae species. In the process of evolution, most of the genes in the six Rosaceae species were in a state of purifying selection. GO and *cis*-acting element analysis revealed that *GRAS* genes were involved in environmental stress and hormone signal transduction pathways. In addition, we focused on the analysis of the expression pattern of *GRAS* genes and the interaction between *GRAS* and *bZIP* genes in woodland strawberry. The results showed that *FvGRAS* genes may had participated in the growth and development of woodland strawberry and played a key role in the response to drought and salt stress. This work laid a foundation for further research on the relationship between abiotic stress and *FvGRAS* genes in woodland strawberry.

## Supplementary Information

**Additional file 1: Fig. S1.** Phylogenetic tree of *GRAS *genes among six Rosaceae species. Red oval means species-specific duplication and red square indicate lineage-specific duplication events.

**Additional file 2: Fig. S2.** Phylogenetic tree of *GRAS *genes in the six Rosaceae species and *Arabidopsis*.

**Additional file 3: Fig. S3.** Mapchart shown the distribution of GRAS genes on 7 chromosomes in woodland strawberry.

**Additional file 4: Fig. S4.** Mapchart shown the distribution of GRAS genes on 17 chromosomes in apple.

**Additional file 5: Fig. S5.** Mapchart shown the distribution of GRAS genes on 8 chromosomes in peach.

**Additional file 6: Fig. S6.** Mapchart shown the distribution of GRAS genes on 17 chromosomes in European pear.

**Additional file 7: Fig. S7.** Mapchart shown the distribution of GRAS genes on 7 chromosomes in Chinese rosa.

**Additional file 8: Fig. S8.** Mapchart shown the distribution of GRAS genes on 7 chromosomes in black raspberry.

**Additional file 9: Fig. S9.** Conserved domain analysis of the *GRAS* gene family among six Rosaceae species.

**Additional file 10: Fig. S10.** Heatmap shown the expression of *bZIP* genes under drought (A) and salt stress (B) in woodland strawberry.

**Additional file 11: Table S1.** Total of 333 GRAS genes were identified in six Rosaceae species. **Table S2.** Ks values of six Rosaceae species. **Table S3.** Duplication events of six Rosaceae species. **Table S4.** ka/Ks values of six Rosaceae species. **Table S5.** Primers used to detect drought stress and salt stress gene expression.

## Data Availability

The datasets supporting the conclusions of this article are included in the article and its Additional files.

## Abbreviations

GAI: Gibberellic acid insensitive
SCR: Scarecrow
RGA: Repressor of ga1-3
STEM: Short Times-series Expression Miner software
FPKM: Fragments per kilobase per million of reads mapped
RT-qPCR: Quantitative real-time PCR
Ka: Nonsynonymous substitution
Ks: Synonymous substitution
PCC: Pearson correlation coefficient
TD: Tandem
WGD: Whole-genome
PD: Proximal
TRD: Transposed
DSD: Dispersed
