# Supplementary material for: Evolution and functional analysis of the GRAS family genes in six Rosaceae species
Source: BMC Plant Biol. 2022 Dec 6;22:569. doi: 10.1186/s12870-022-03925-x (PMC9724429; doi:10.1186/s12870-022-03925-x)
Supplement: Supplementary file 1 — Additional file 1: Fig. S1. Phylogenetic tree of GRAS genes among six Rosaceae species. Red oval means species-specific duplication and red square indicate lineage-specific duplication events. [file 12870_2022_3925_MOESM1_ESM.pdf]

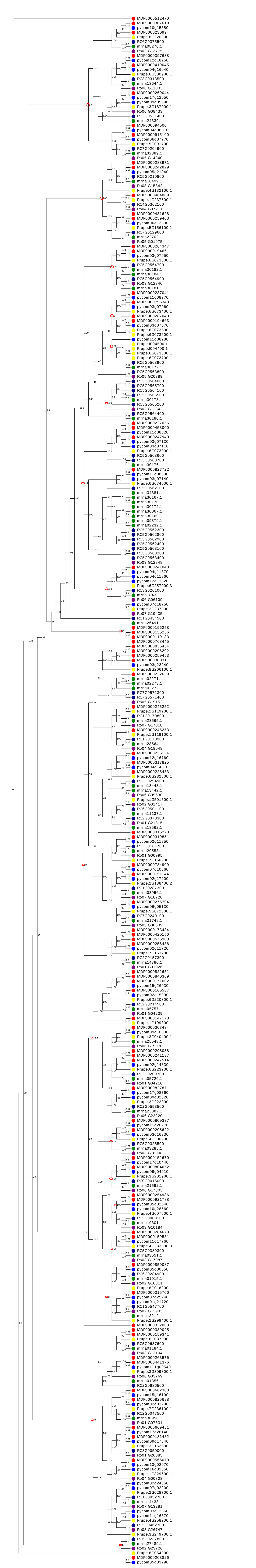

Fig. S1 Phylogenetic tree of *GRAS* genes among six Rosaceae species. Red oval means species-specific duplication and red square indicate lineage-specific duplication events.
