# Supplementary figures and images for: Evolution and functional analysis of the GRAS family genes in six Rosaceae species
Source: BMC Plant Biol. 2022 Dec 6;22:569. doi: 10.1186/s12870-022-03925-x (PMC9724429; doi:10.1186/s12870-022-03925-x)

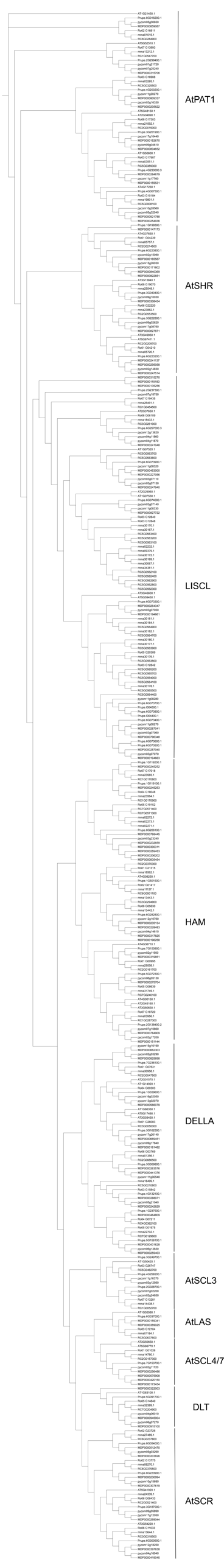

Supplement: Supplementary file 2 — Additional file 2: Fig. S2. Phylogenetic tree of GRAS genes in the six Rosaceae species and Arabidopsis. [file 12870_2022_3925_MOESM2_ESM.pdf]

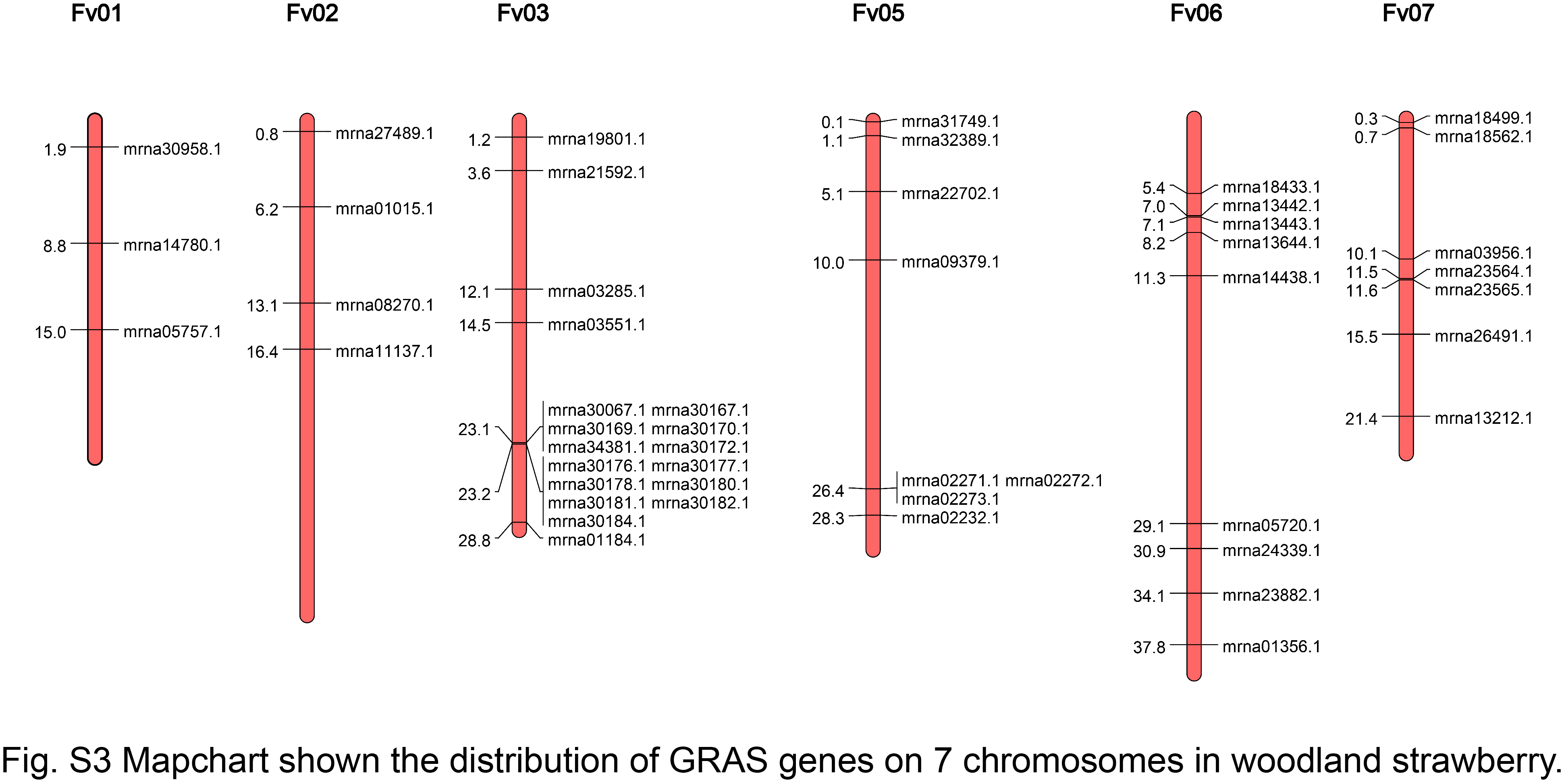

Supplement: Supplementary file 3 — Additional file 3: Fig. S3. Mapchart shown the distribution of GRAS genes on 7 chromosomes in woodland strawberry. [file 12870_2022_3925_MOESM3_ESM.jpg]

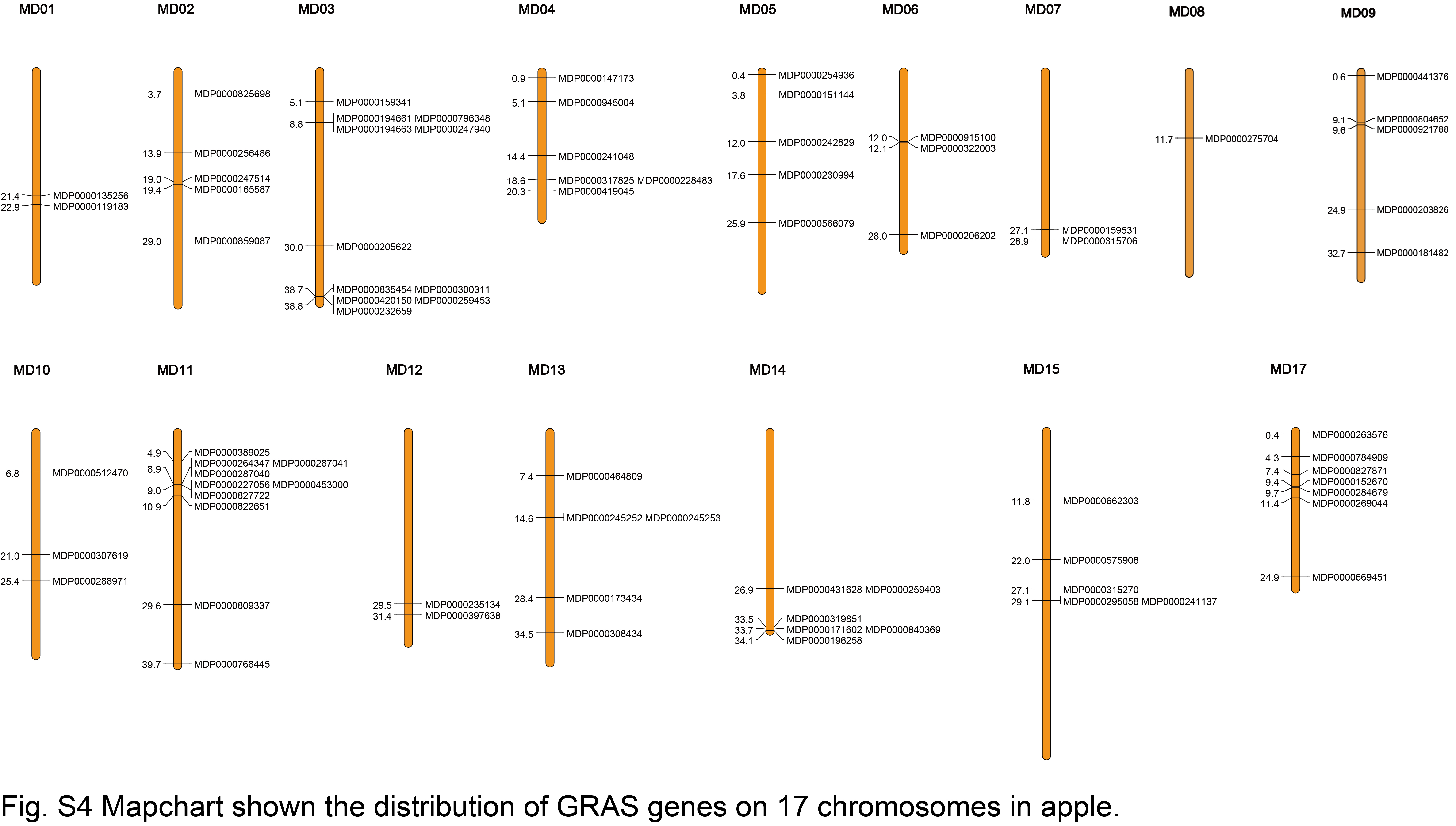

Supplement: Supplementary file 4 — Additional file 4: Fig. S4. Mapchart shown the distribution of GRAS genes on 17 chromosomes in apple. [file 12870_2022_3925_MOESM4_ESM.jpg]

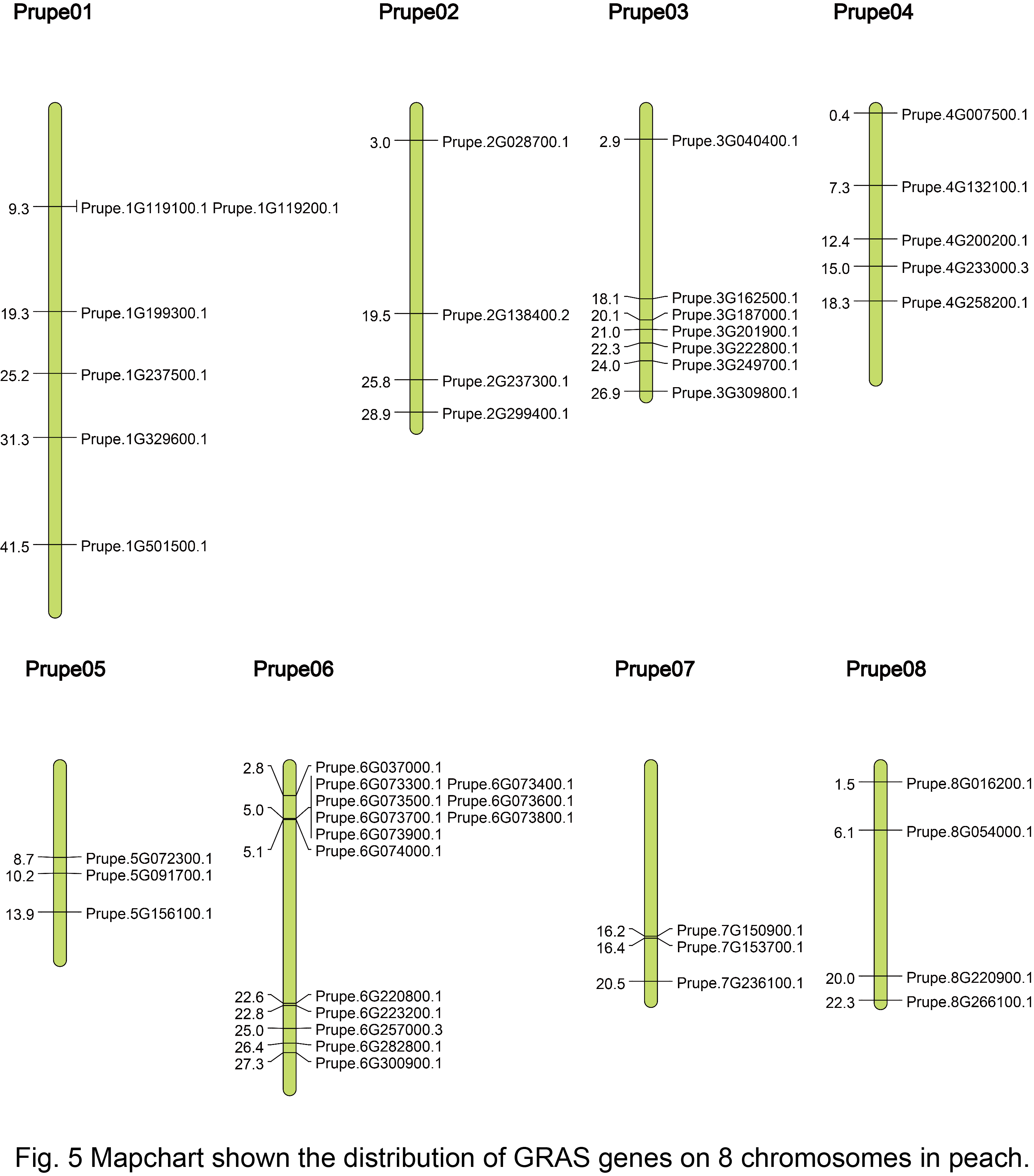

Supplement: Supplementary file 5 — Additional file 5: Fig. S5. Mapchart shown the distribution of GRAS genes on 8 chromosomes in peach. [file 12870_2022_3925_MOESM5_ESM.jpg]

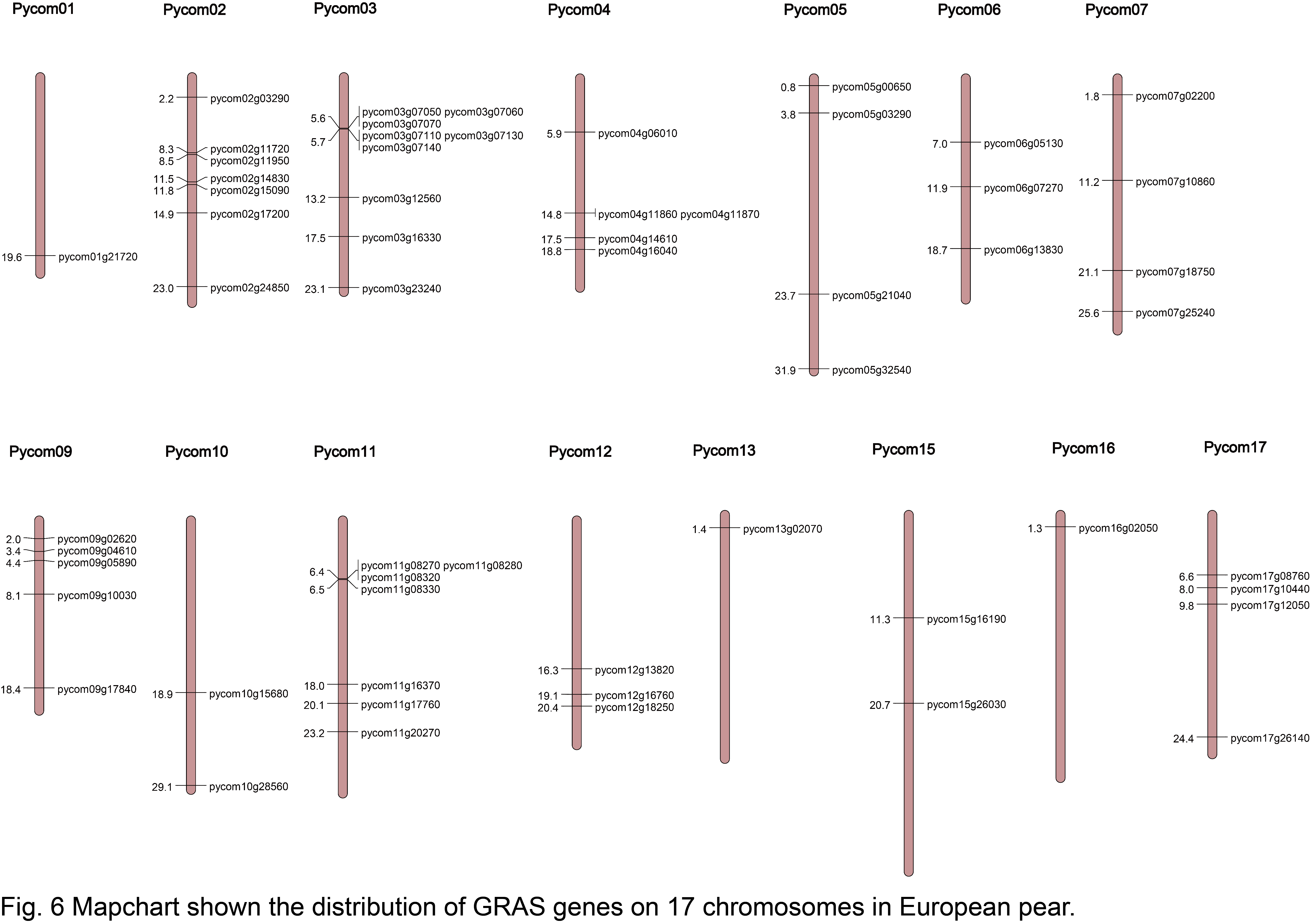

Supplement: Supplementary file 6 — Additional file 6: Fig. S6. Mapchart shown the distribution of GRAS genes on 17 chromosomes in European pear. [file 12870_2022_3925_MOESM6_ESM.jpg]

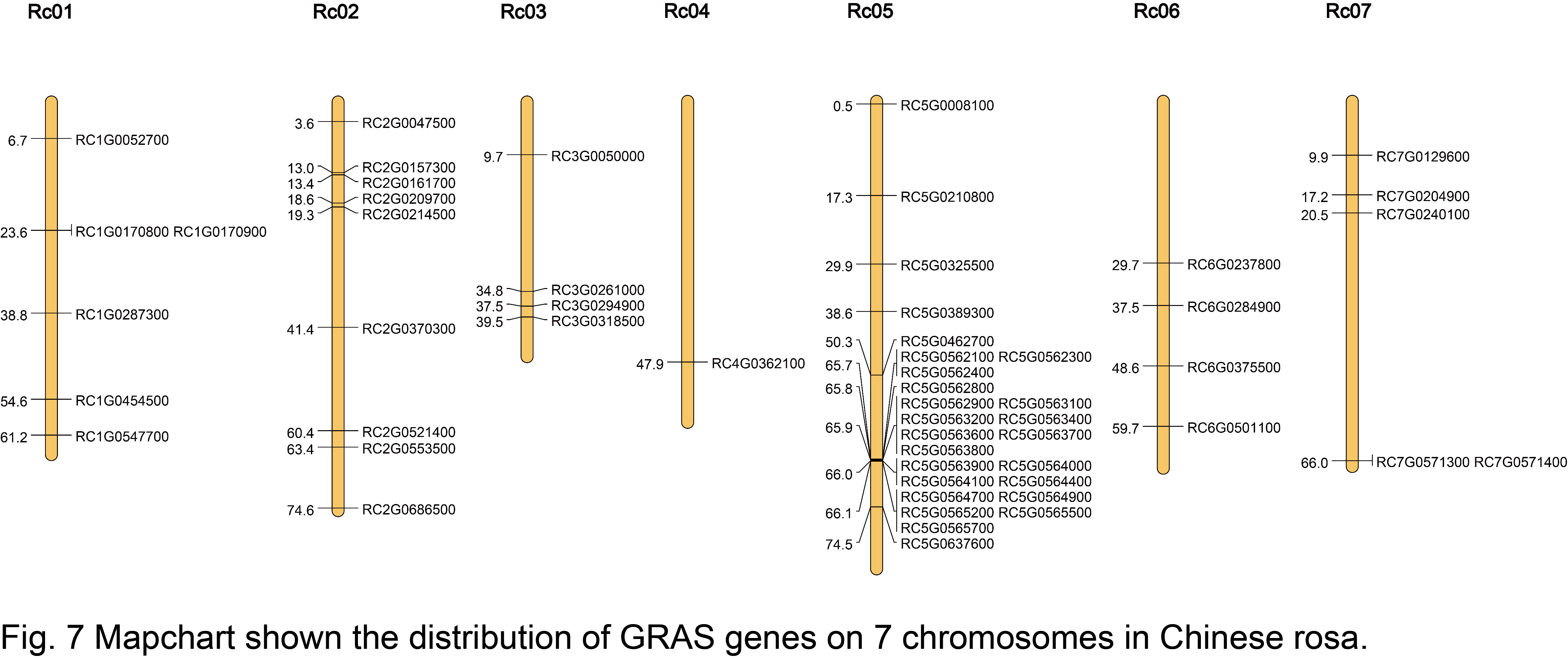

Supplement: Supplementary file 7 — Additional file 7: Fig. S7. Mapchart shown the distribution of GRAS genes on 7 chromosomes in Chinese rosa. [file 12870_2022_3925_MOESM7_ESM.jpg]

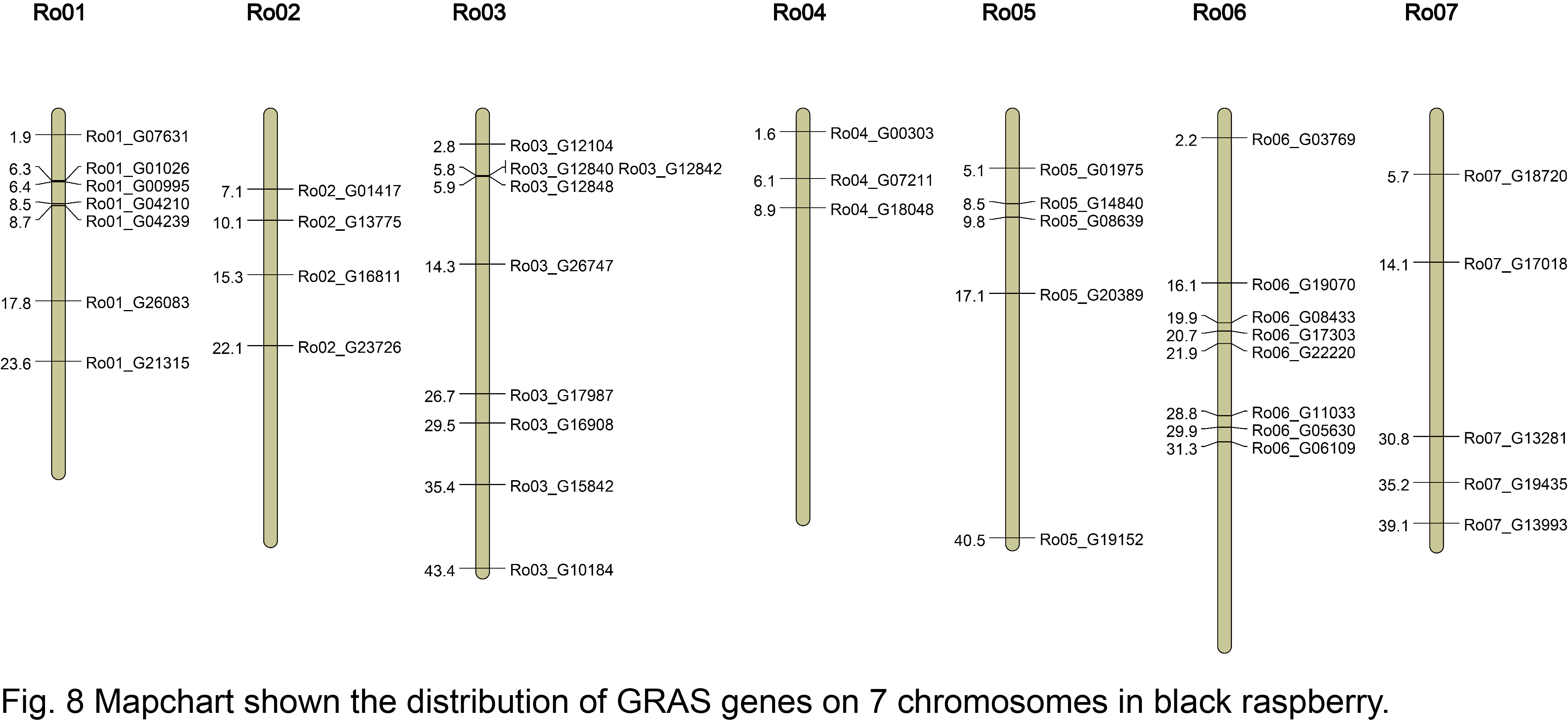

Supplement: Supplementary file 8 — Additional file 8: Fig. S8. Mapchart shown the distribution of GRAS genes on 7 chromosomes in black raspberry. [file 12870_2022_3925_MOESM8_ESM.jpg]

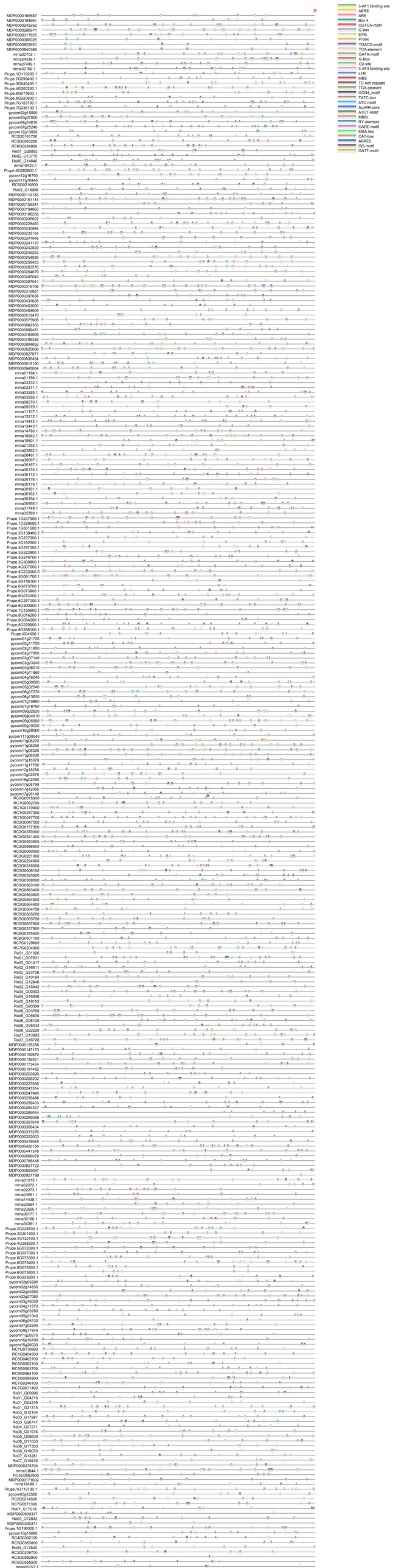

Fig. S9 Conserved domain analysis of the GRAS gene family among six Rosaceae species.

Supplement: Supplementary file 9 — Additional file 9: Fig. S9. Conserved domain analysis of the GRAS gene family among six Rosaceae species. [file 12870_2022_3925_MOESM9_ESM.pdf]
