# Supplementary material for: Evolution and functional analysis of the GRAS family genes in six Rosaceae species
Source: BMC Plant Biol. 2022 Dec 6;22:569. doi: 10.1186/s12870-022-03925-x (PMC9724429; doi:10.1186/s12870-022-03925-x)
Supplement: Supplementary file 10 — Additional file 10: Fig. S10. Heatmap shown the expression of bZIP genes under drought (A) and salt stress (B) in woodland strawberry. [file 12870_2022_3925_MOESM10_ESM.pdf]

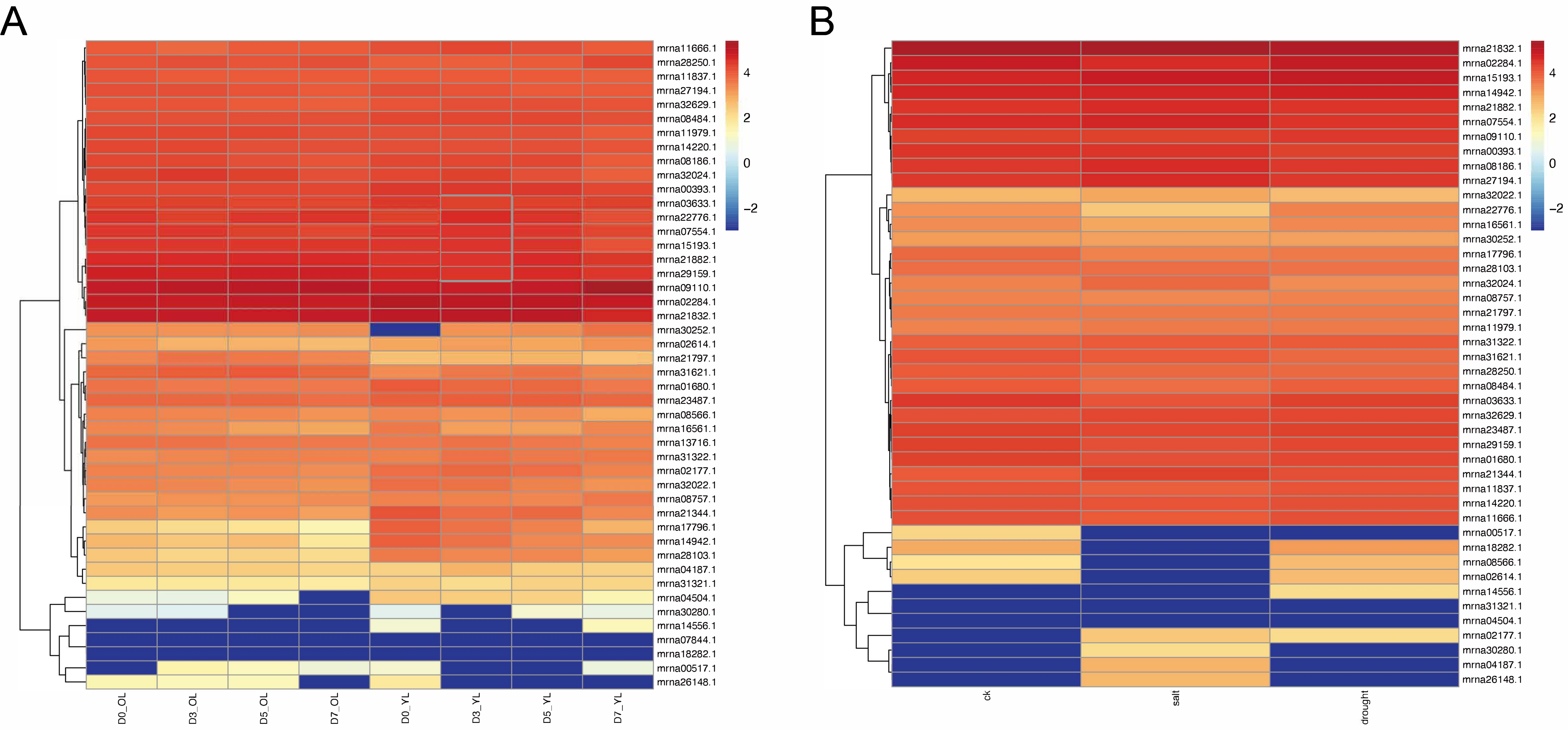

Fig. S10 Heatmap shown the expression of *bZIP* genes under drought (A) and salt stress (B) in woodland strawberry.
